# Postoperative Probiotics Administration Attenuates Gastrointestinal Complications and Gut Microbiota Dysbiosis Caused by Chemotherapy in Colorectal Cancer Patients

**DOI:** 10.3390/nu15020356

**Published:** 2023-01-11

**Authors:** Feng Huang, Shengjie Li, Wenjie Chen, Yiyang Han, Yue Yao, Liang Yang, Qiang Li, Qun Xiao, Jing Wei, Zhaoxia Liu, Tingtao Chen, Xiaorong Deng

**Affiliations:** 1 Department of Gastrointestinal Surgery, The Second Affiliated Hospital of Nanchang University, Nanchang 330006, China; 2 Institute of Translational Medicine, Nanchang University, Nanchang 330031, China; 3 Department of Vascular Surgery, Tengzhou Central People’s Hospital, Zaozhuang 277599, China; 4 Department of Hepatobiliary Pancreatic Splenic Surgery, The First Affiliated Hospital of Hunan College of Traditional Chinese Medicine, Zhuzhou 410208, China; 5 Department of Obstetrics and Gynecology, The Second Affiliated Hospital of Nanchang University, Nanchang 330006, China

**Keywords:** probiotics, CRC, chemotherapy, gastrointestinal complications, gut microbiota

## Abstract

The current study aims to evaluate the potential roles of taking probiotics postoperatively in attenuating the gastrointestinal complications and disturbed gut microbiota in colorectal cancer (CRC) patients undergoing chemotherapy. One hundred eligible CRC patients who were treated with radical surgery and needed to receive chemotherapy were recruited. Half of them were randomly assigned to the Probio group to take a probiotic combination from post-operation to the end of the first chemotherapeutic course. The other half of patients taking placebo instead were classified as the Placebo group. Gastrointestinal complications such as nausea, acid reflux, abdominal pain, abdominal distention, constipation, and diarrhea were recorded during chemotherapy. Fecal samples were collected preoperatively and after the first cycle of postoperative chemotherapy for 16S rRNA high-throughput sequencing and short-chain fatty acids (SCFAs) analysis. Results showed that probiotics administration could effectively reduce chemotherapy-induced gastrointestinal complications, particularly in diarrhea (*p* < 0.01). Additionally, chemotherapy also reduced the bacterial diversity indexes of the gut microbiota in CRC patients, which could be significantly increased by taking probiotics. Moreover, this chemotherapy caused significant changes in the composition of the gut microbiota, as indicated by decreased phylum levels of Firmicutes and increased Bacteroidetes, Proteobacteria, and Verrucomicrobia. In particular, several bacterial genera such as *Akkermansia* and *Clostridium* were significantly increased, while *Prevotella*, *Lactobacillus*, and *Roseburia* were decreased (*p* < 0.05). However, probiotic administration could effectively restore these taxa changes both at the phylum and genus levels, and mildly increase the genus levels of *Bifidobacterium*, *Streptococcus*, and *Blautia*. Furthermore, probiotics could also promote the production of SCFAs, particularly increasing acetate, butyrate, and propionate (*p* < 0.0001). These results support the beneficial effects of the probiotic interventions as novel alternative or complementary strategies in chemoprevention.

## 1. Introduction

Colorectal cancer (CRC) is one of the world population’s most common malignant tumors of the digestive tract. Statistical data based on epidemiological status shows that the incidence of CRC firmly places it in the third spot for the most common cancer, and the mortality rate ranks fourth for the most common cause of death in cancers [1]. Thus, CRS has become the most threatening killer for human life and health. Most CRC cases were diagnosed at the late stage and accompanied by distal organ metastasis since no obvious clinical manifestations were observed in their early stage, leading to the loss of the best opportunity for radical surgery [2,3]. Although the multidisciplinary strategies based on surgery center were effective in treating CRC, their efficacy is still unsatisfactory, as indicated by many complications observed in the therapeutic process, including postoperative infections, diarrhea, abdominal pain, nausea and vomiting, and even gastrointestinal bleeding, particularly in the adjuvant chemotherapy [4,5].

The common adjuvant chemotherapy can be a double-edged sword. On the one hand, it plays a crucial role in the control and treatment of cancers such as CRC by inhibiting both tumor growth and cancer development in CRC [1,6]; on the other hand, it brings the recognized different degrees of cell toxicity and side effects, which in turn limit the anticancer efficacy of chemotherapeutic drugs and affect the patient’s quality of life [5,7]. In particular, increasing evidence suggests that there is a disturbance of gut microbiota (also known as gut dysbiosis) in CRC patients receiving chemotherapy, and a positive correlation between gut dysbiosis and the risk of CRC recurrence [8,9,10], indicating that gut dysbiosis might be an independent risk factor for the recurrence of CRC following chemotherapy [11]. Moreover, short-chain fatty acids (SCFAs), the principal productions generated from gut microbial metabolism, were also changed in the intestine of CRC subjects after anticancer therapy, as indicated by decreasing acetate, butyrate, and propionate [12,13]. Therefore, seeking new alternative or complementary strategies to slow down the side effects of chemotherapy and remodel the gut microbiota is of great interest.

Probiotics, live microorganisms characterized by their health-promoting benefits in improving gut ecosystems, enhancing immunity, suppressing tumors, and inhibiting inflammation, have attracted our attention to solving the abovementioned problems. We have demonstrated that different probiotic combinations could reduce the inflammatory responses at the site of tumor genesis, enhance the immunity of patients, restore gut functions, and alleviate the side effects of anticancer treatments in various tumors via remodeling the disturbed gut microbiota [14,15,16,17,18], supporting the beneficial effects of probiotics for the prevention and treatment of cancers. Here, we performed a clinical study to explore the potential roles of a probiotic combination containing *Bifidobacterium infants*, *Lactobacillus acidophilus*, *Enterococcus faecalis*, and *Bacillus cereus*, in attenuating the gastrointestinal complications and gut dysbiosis of CRC patients who underwent chemotherapy. The observed data suggest that probiotic administration based on gut microbiota may be effective for CRC patients during postoperative clinical management.

## 2. Materials and Methods

### 2.1. Probiotic and Placebo Tablets

Combined *B. infants*, *L. acidophilus*, *E. faecalis*, and *B. cereus* tablets (live) involved in this study were manufactured by Hangzhou Grand Biologic Pharmaceutical INC, Hangzhou, China [14]. Each tablet weighed 0.5 g and contained over 0.5 × 10^6^ CFU of *B. infants*, *L. acidophilus*, *E. faecalis*, and over 0.5 × 10^5^ CFU of *B. cereus*, respectively. The probiotic tablets have been approved as biological products by China Food and Drug Administration with a batch number of S20060010. Placebo tablets without any live probiotics or microorganisms were similar to the probiotic ones in terms of taste and texture, and were prepared by the same company. All tablets were kept at 2–8 °C temperature and shielded from light.

### 2.2. Participants

A randomized, single-blind, placebo-controlled prospective study was conducted at the Second Affiliated Hospital of Nanchang University, China, in the period from April 2021 to April 2022. All procedures were reviewed and approved by the Institutional Ethics Committee on medical research of this hospital. The study was also registered to the base of clinical trials, and the registration identifier was ChiCTR2000040916.

A total of 100 patients diagnosed with CRC, satisfying the inclusion and exclusion criteria, were recruited finally. All participants signed the informed consents before enrolment, and their privacy was protected. The inclusion criteria included: (1) patients aged 40–70 years old with pathohistologically proven CRC, needing to undergo surgical resection and receive chemotherapy subsequently; (2) patients who did not have accompanying severe heart, lung, kidney, and liver dysfunctions and metabolic diseases; (3) patients with no distant metastases found. The exclusion criteria were as follows: (1) individuals had used antibiotics or consumed pro/pre/syn-biotic products within two weeks; (2) patients with metabolic diseases (e.g., diabetes and diabetes) and severe concomitant disorders in heart, lung, liver or kidneys; (3) patients with a history of inflammatory bowel disease and colorectal adenoma; (4) individuals with a family history of CRC and other gastrointestinal cancers; (5) other factors, such as hypersensitivity to study drugs and pregnant women.

### 2.3. Treatment Procedure

**Figure 1:**
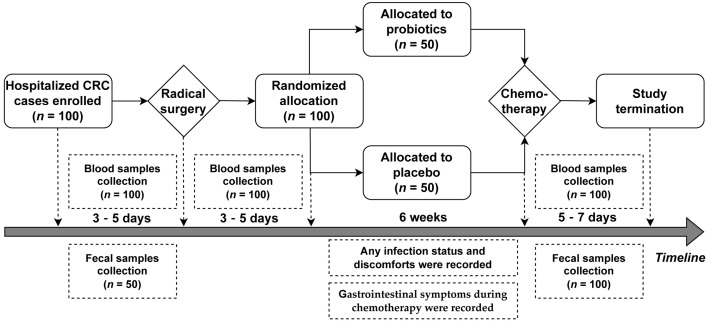
Flow diagram showing the schedule of the study.

The eligible patients were randomly classified (in a ratio of 1:1) into two groups: the Placebo group and the Probio group, after receiving surgical resection treatment. Patients in the Probio group were instructed to take probiotic tablets orally according to the scheme 1 × 3 tablets (one capsule at a time and three times per day) from the third postoperative day to the end of the first chemotherapy course (Capecitabine **+** Oxaliplatin, XELOX regimen). Patients in the Placebo group were routinely treated with placebo tablets instead of probiotics. The time of intervention duration was about six weeks, containing two weeks of chemotherapy. Patient gastrointestinal symptoms during the two-week chemotherapeutic period were recorded, including any reported cases of nausea, acid reflux, abdominal pain, abdominal distention, constipation, and diarrhea. Any infection status within six weeks of intervention was recorded, such as acute gastroenteritis and other infections requiring antibiotic treatments. Blood samples were taken from the recruited participants to undergo routine analysis before surgery, upon the first postoperative day, and before the start of and after completing the first chemotherapeutic course. Fifty feces samples from fifty randomized subjects were randomly collected as the standard reference samples when they were recruited (*n* = 50), as well as collected from all patients both in the Probio group (*n* = 50) and Placebo group (*n* = 50) at the end of this study. All fecal samples were stored at −80 °C for further analysis. The schedule of the treatment procedure and sample collection is shown in Figure 1.

### 2.4. Microbial DNA Extraction and 16S rRNA Sequencing Analysis

Total microbial genomic DNA was extracted from all fecal samples using TIANamp Stoll DNA Kit (TIANGEN Biotech Co., Ltd., Beijing, China; Catalog No.: DP328) according to the manufacturer’s protocol. The quality and concentration of total extracted DNA were identified by 1.5% agarose gel electrophoresis and Nanodrop spectrophotometer (Thermo Fisher Scientific, Inc., MA, USA). The subsequent PCR amplification of the V4 region of 16S rDNA, sequencing library construction, and high-throughput sequencing (Illumina NovaSeq/MiSeq platform) were performed at Shanghai Personalbio Technology Co., Ltd. (Shanghai, China). The raw reads were deposited in the Sequence Read Archive (SRA) database of NCBI (PRJNA903224).

Microbiome bioinformatics were performed with QIIME 2 2019.4 [19] with slight modifications according to the official tutorials (https://docs.qiime2.org/2019.4/tutorials/, accessed on 14 April 2022). Briefly, raw sequence data were demultiplexed using the demux plugin, followed by primers cutting with the cutadapt plugin. Sequences were then quality filtered, denoised, merged, and chimera removed using the DADA2 plugin [20]. The effective reads were clustered according to sequence similarity, and sequences with ≥97% similarity were assigned to the same operational tax units (OTUs). The common and unique OTUs of each group were analyzed and compared. The OTU sequences were compared by SILVA software (https://www.arb-silva.de/, accessed on 14 April 2022) and further annotated. Alpha-diversity metrics such as Chao1 (species richness), Simpson (bacterial diversity), and Pielou’s evenness (PE) were calculated using the diversity plugin with samples. Principal coordinate analysis (PCoA) analysis was conducted based on the unweighted UniFrac and Jaccard distance to observe the different degrees of microbial community structure among samples, which was the Beta-diversity analysis. Linear discriminant analysis (LDA) effect size (LEfSe) was used to identify the relative abundance of bacterial taxa in different groups.

### 2.5. Detection of Short Chain Fatty Acids (SCFAs)

The method of sample preparation and extraction was described previously [21,22]. Briefly, 20 mg of fecal samples were accurately weighed and placed in a 2 mL EP tube. Then, 1 mL of phosphoric acid (0.5% *v*/*v*) solution and a small steel ball were added to the EP tube. The samples were ground uniformly, then vortexed for 10 min, and ultrasonicated for 5 min. Then, 0.1 mL of supernatant was added to the 1.5 mL centrifugal tube after the mixture was centrifuged at 12,000 rpm for 10 min at the temperature of 4 °C. Following this, 0.5 mL MTBE (containing internal standard) solution was added to the centrifugal tube. The mixture was vortexed for 3 min and ultrasonicated for 5 min. After that, the mixture was centrifuged at 12,000 r/min for 10 min at the temperature of 4 °C. The supernatant was collected and used for GC-MS/MS analysis. All chemicals, standards, and reagents were purchased from CNW (CNW Technologies, Germany) and Aladdin (Shanghai, China).

SCFAs contents were detected by MetWare (http://www.metware.cn/, accessed on 17 August 2022) based on the Agilent 7890B gas chromatograph coupled to a 7000D mass spectrometer with a DB-FFAP column (30 m length × 0.25 mm i.d. × 0.25 μm film thickness, J&W Scientific, USA). Helium was used as the carrier gas at a flow rate of 1.2 mL/min. The injection was made in the split mode, and the injection volume was 2 μL. The oven temperature was held at 90 °C for 1 min, raised to 100 °C at a rate of 25 °C/min, then raised to 150 °C at a rate of 20 °C/min, held for 0.6 min, raised to 200 °C at a rate of 25 °C/min, held for 0.5 min, after running for 3 min. All samples were analyzed in multiple reaction monitoring modes. The injector inlet and transfer line temperatures were 200 °C and 230 °C, respectively [22,23].

### 2.6. Statistical Analysis

Statistical analysis was performed using Prism software version 8.0 (GraphPad Software, San Diego, CA, USA). Data were shown as the mean ± SD. Statistical significance was analyzed using Fisher’s exact test and one-way analysis of variance (ANOVA) followed by Tukey’s multiple comparison test and the Kruskal–Wallis nonparametric test. Error probabilities of *p* < 0.05 were considered statistically significant.

## 3. Results

### 3.1. Clinical Characteristics of CRC Patients

Patients’ clinical characteristics, including age, gender, tumor/node/metastasis category (TNM category), tumor pathological subtype and site, were identified (Table 1). There was no statistical difference in the patients’ gender and age with the majority being in their 60s. Regarding the cancer category, most patients were diagnosed with TNM II and III stages, but no significant difference was observed between the two groups. In addition, no patients with metastatic CRC were recruited in this study. Similarly, the majority of tumor locations were at the site of the colon. Thus, these clinical records suggested that the recruited patients were homogeneous between these two groups.

### 3.2. Probiotics Improve Gastrointestinal Complications Induced by Chemotherapy

No infection cases or required antibiotic administrations among participants were recorded during the six-week intervention period. There is no statistical difference in most physiological indexes of blood routine analysis before surgery and on the first postoperative day, except for decreased neutrophil and white blood cell counts, and increased albumin after surgery (Table S1). Interestingly, twelve out of fifty patients in the Probio group and three out of fifty patients in the Placebo group complained of abdominal pain, which led to a remarkable difference in statistics (*p* = 0.025) (Table 2). In addition, there were similar variation trends in the number of patients who had abdominal distention (*p* = 0.041) and constipation (*p* = 0.019) during the chemotherapeutic period. It was noticed that only eight patients in the Probio group reported having the reaction type of diarrhea, which was dramatically lower than that of patients in the Placebo group (*p* = 0.008). However, no significant differences were observed in the blood routine indexes among the two groups’ patients undergoing chemotherapy (Table S1), indicating that taking probiotics might not alter the treatment efficacy of XELOX regimen-based chemotherapy. These findings suggested that probiotic administration could effectively reduce chemotherapy-induced gastrointestinal complications, especially in the case of diarrhea, without affecting the antitumor efficacy of chemotherapy.

### 3.3. Probiotics Remodel the Disturbed Intestinal Bacterial Diversity

**Figure 2:**
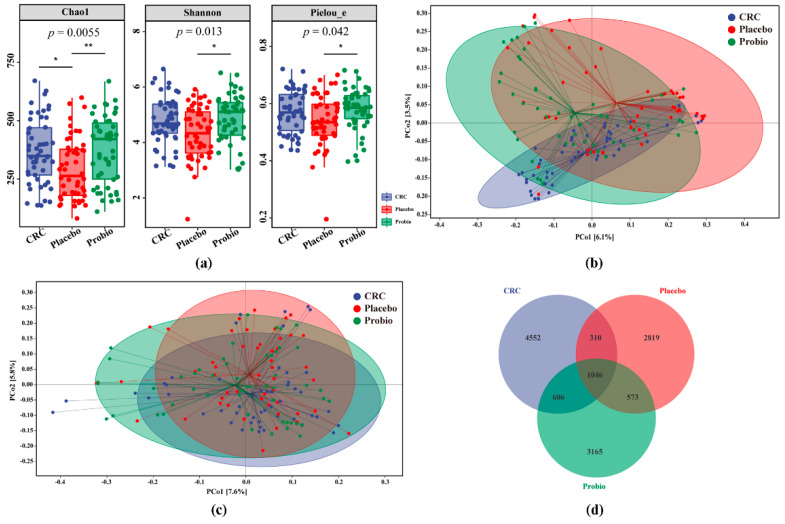
Probiotics combination restored the changed gut bacterial diversity in CRC patients receiving chemotherapy. (**a**) Chao1, Simpson, and Pielou_e (PE) indexes of gut microbial α diversity in the fecal samples among the three groups. (**b**,**c**) The PCoA analysis of gut microbial β diversity based on Jaccard and Unweighted_unifrac distances, respectively. (**d**) Venn diagram of the identified bacterial species among CRC patients. CRC, the standard reference group before anticancer treatment. Placebo, the control group taking placebo. Probio, the treatment group taking probiotic combination. Multiple comparison analysis based on Kruskal−Wallis test following Dunn’s test, * *p* < 0.05, ** *p* < 0.01.

Alpha diversity analysis results showed that the Chao 1 index of the Placebo group was obviously lower than that of the CRC group (*p* < 0.05), while no significant differences were observed in the Shannon (*p* > 0.05) and PE indexes (*p* > 0.05) between the two groups, even though they were comparatively lower in the Placebo group (Figure 2a). Although slightly higher values were observed among these indexes in the Probio group compared to those in the CRC group, the statistical differences were insignificant (Figure 2a). Remarkably, these indexes of the Probio group were significantly increased when compared with that of the Placebo group (*p* < 0.05), particularly in the Chao1 index (*p* = 0.0055). The changing trend of observed species was similar to that of the Chao1 index (Figure S1a), and the value of Good’s coverage for each sample was over 99.8% (Figure S1a).

The results of PCoA analysis, based on the Jaccard distance, revealed that there was a mild separation among the three groups, although samples from all groups mostly overlapped with one another, indicating the distance of samples was consistent with the grouping (Figure 2b). However, there were no clear distinctions for the fecal microbial communities among all groups, based on the analysis of Unweighted_unifac distance, due to most of samples overlapping with each other (Figure 2c). The change in beta diversity based on Bray_curtis and Weighted_unifac was also similar to that of the Unweighted_unifac (Figure S1b,c). Furthermore, a total of 13,071 OTUs were generated from those sequenced samples following quality control, in which 1046 OTUs were shared among all groups, while the order of the total number of OTUs or fecal bacteria in each group was CRC > Probio > Placebo (Figure 2d). These observations suggested that surgery combined with chemotherapy for CRC would disturb the gut microbiota diversity, but probiotic treatment could alleviate this changing trend.

### 3.4. Probiotics Restore the Changed Gut Bacterial Taxa

**Figure 3:**
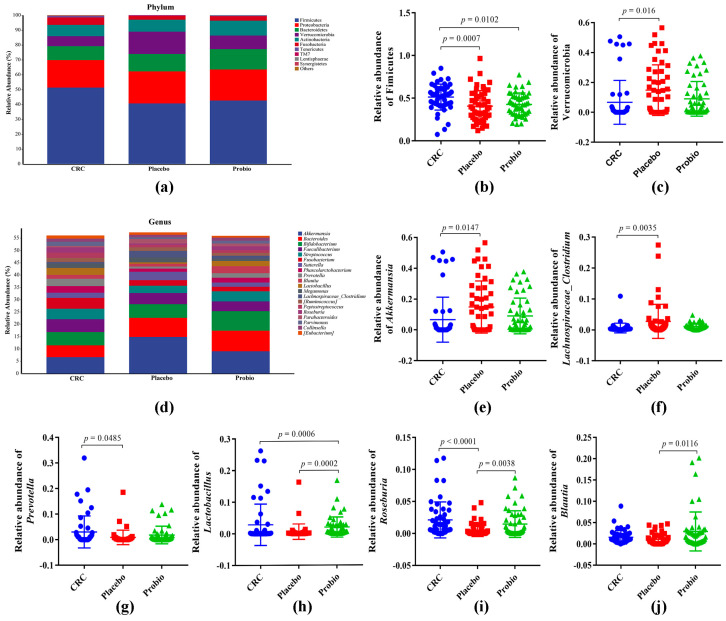
Probiotics combination remodeled the different gut microbial taxa among CRC patients. (**a**) Stacking map of taxa distribution at the phylum level. (**b**,**c**) The relative abundance of Firmicutes and Verrucomicrobia at the phylum level. (**d**) Stacking map of species distribution at the genus level. (**e**,**f**) The relative abundance of *Akkermansia* (**e**), *Lachnospiraceae_Clostridium* (**f**), *Prevotella* (**g**), *Lactobacillus* (**h**), *Roseburia* (**i**), and *Blautia* (**j**). CRC, the standard reference group before anticancer treatment. Placebo, the control group taking placebo. Probio, the treatment group taking probiotics combination. One−way ANOVA Multiple comparision was based on Kruskal−Wallis test following Dunn’s test. *p* value < 0.05 means statistical significance.

The phylum of Firmicutes, Proteobacteria, Bacteroidetes, Verrucomicrobia, and Actinobacteria constituted over 90% of bacterial phyla in each group (Figure 3a). Among them, the relative abundance of Firmicutes in the intestinal microflora of CRC patients was reduced from 51.33% in the CRC group to 40.69% in the Placebo group (Figure 3b), as well as Fusobacteria reduced from 4.99% to 2.56% (Figure 3a). In contrast, the abundance of Bacteroidetes, Proteobacteria, and Verrucomicrobia increased in the fecal samples of CRC patients following radical surgery and chemotherapy, suggesting that the chemical treatment of CRC changed the gut bacterial taxa at the phylum level. Moreover, probiotic administration clearly reversed the changing trend of Verrucomicrobia, but hardly affected the abundance of other bacterial phyla. At the top 20 genera level (Figure 3d), the relative abundance of *Akkermansia* (*p* = 0.0147) and *Lachnospiraceae_Clostridium* (*p* = 0.0035) in the Placebo group was significantly higher than that in the CRC group (Figure 3e,f), while that of *Prevotella*, *Lactobacillus*, and *Roseburia* was obviously lower (*p* < 0.05) (Figure 3g–i). Interestingly, taking probiotics could effectively restore these changed genera to the standard level, as well as increase the relative abundance of *Bifidobacterium*, *Streptococcus*, and *Blautia* (compared to that of the Placebo group, *p* = 0.0116, Figure 3j). Furthermore, it also decreased the relative abundance of *Faecalibacterium*, *Fusobacterium*, *Sutterella*, and *Megamonas* compared with that of the Placebo group (Figure 3d).

**Figure 4:**
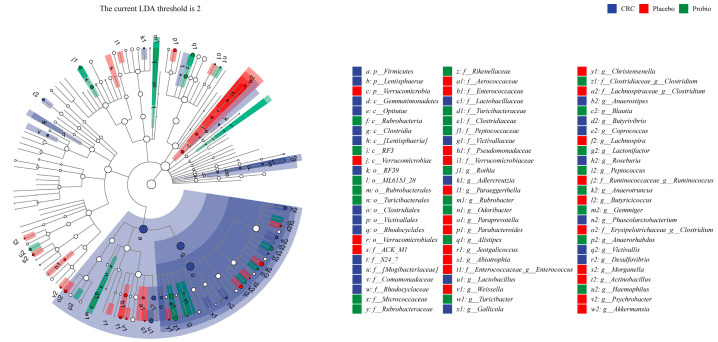
LEfSe cladogram showing differently abundant gut microbiota taxa among CRC patients at different levels. The current LDA threshold score is over 2; *p*, phylum; *c*, class; *o*, order; *f*, family; *g*, genus. The blue, red, and green color refers to different bacterial taxa in CRC group, Placebo, and Probio group, respectively.

LEfSe analysis showed that the gastrointestinal tract of CRC patients was enriched with Firmicutes phylum, *Clostridia* class, *Clostridiales* order, *Lactobacillaceae* family, *Roseburia* genus, *Phascolarctobacterium* genus, *Lactobacillus* genus, and *Desulfovibrio* genus (Figure 4). In addition, the GI tract of CRC patients who underwent surgery and chemotherapy (Placebo group) was enriched with Verrucomicrobia phylum to *Akkamansia* genus, *Enterococcaceae* family to *Enterococcus* genus, *Clostridium* genus, *Parabaceroides* genus, and *Ruminococcus* genus. Compared with the Placebo group, the gut microflora of CRC patients in the Probio group was enriched with *Blautia*, *Haemohilus*, *Alistipes*, *Gemmiger* and *Clostridium* genera, and *Rikenellaceae* and *Clostrdiaceae* families. Thus, these results indicated that surgical and chemical therapy cause significant changes to the composition of the gut microbiota of CRC patients, but probiotic administration during the treatment period could reshape the disturbed gut bacterial populations.

### 3.5. Probiotics Increase the Production of Intestinal SCFAs

**Figure 5:**
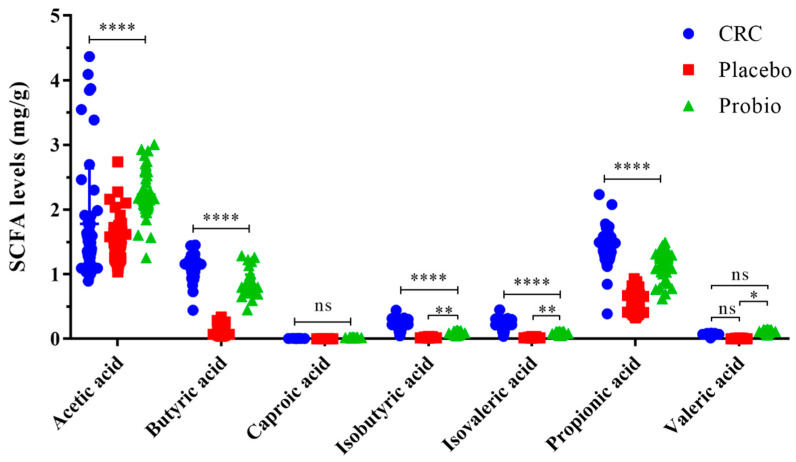
Probiotics combination promoted the production of SCFAs in the gut of CRC patients undergoing chemotherapy. CRC, the standard reference group before anticancer treatment. Placebo, the control group taking placebo. Probio, the treatment group taking probiotics combination. Multiple comparisons based on two-way ANOVA analysis following Tukey’s test, * *p* < 0.05, ** *p* < 0.01, **** *p* < 0.0001; ns refers to no significant difference detected. The capped line refers to comparison among the three groups; the half tick-down line refers to comparison between the two groups.

The concentrations of SCFAs in the fecal samples of CRC patients were shown in Figure 5. The main SCFAs detected in all samples were acetic acid, propionic acid, and butyric acid, with an acknowledged content order acetic acid > propionic acid > butyric acid. In addition, small contents of isobutyric acid, valeric and isovaleric acid, and caproic acid were detected as well. Noticeably, chemical treatment based on the XELOX regimen significantly decreased the levels of acetic acid, propionic acid, butyric and isobutyric acid, and isovaleric acid (CRC vs. Placebo, *p* < 0.0001), but had no impacts on that caproic acid and valeric acid (*p* > 0.05). Comfortingly, probiotic intervention before and during the chemotherapy period dramatically increased the levels of the main SCFAs compared to the Placebo groups (*p* < 0.0001). In addition, probiotics hardly reversed the changes in terms of caproic acid, isobutyric acid, and isovaleric acid (*p* > 0.05), with the exception of valeric acid (*p* = 0.0188). The association of the differentially abundant bacterial genera with the levels of fecal SCFAs was explored using Spearman correlation analysis (Figure S2). The increased *Phascobarctobacterium*, *Lactobacillus*, and *Roseburia* genera in the Probio group had a significant positive correlation with the fecal almost SCFAs. In addition, the decreased *Akkemansia* and *Sutterella* seemed to be negatively correlated to these SCFAs. These results indicated that probiotics administration could increase the production of SCFAs in the intestine of CRC patients who underwent chemotherapy, which was associated with reshaped gut bacterial populations.

## 4. Discussion

Growing clinical and animal studies have evidenced that chemotherapeutic drugs can cause various side effects during anticancer treatment periods, including the destruction of the immune system, the induction of gastrointestinal mucosal inflammation, the dysbiosis of gut microbiota, as well as the injuries of physiological functions [5]. These chemotherapy-induced complications can adversely impact the chemotherapy’s anticancer efficacy, leading to treatment delays and even therapy failure. Fortunately, several pharmacological or nonpharmacological interventions (e.g., probiotics) have been suggested to attenuate chemotherapy-mediated symptoms in CRC subjects. In this study, we demonstrated that XELOX regimen-based chemotherapy would induce gastrointestinal symptoms such as abdominal distention, abdominal pain, constipation, diarrhea in CRC patients, and gut microbiota dysbiosis. Subsequently, probiotic administration postoperatively could significantly reduce these chemotherapy-induced complications, supporting that the probiotic intervention, based on gut microbiota, is beneficial for CRC patients during postoperative clinical treatment.

To date, many publications have reported the promising preventive strategy of probiotics in decreasing the incidence of chemotherapy-induced gastrointestinal complications in CRC patients [24,25]. According to the published results, using probiotics—ignoring the probiotic type and formula—preoperatively or perioperatively for patients with CRC, could improve the clinical outcomes of radical surgery and adjuvant chemotherapy, particularly regarding adverse gastrointestinal effects such as diarrhea and abdominal discomfort [26,27]. In our previous study, a probiotic combination containing four different species, i.e., *B. infants*, *L. acidophilus*, *E. faecalis*, and *B. cereus*, had been proven to significantly reduce partial gastrectomy-mediated physiology and gut microbial disorders in gastric cancer [14]. Here, we further expanded on the application of this probiotic combination in adjuvant chemotherapy for CRC (Table 2), adding new evidence for using probiotics, and their clinical significance.

It is proposed that the gastrointestinal pathological injuries following chemotherapy-based treatment may cause dysbiosis of gut microbiota, as indicated by an increase in harmful microbial populations and a decrease in beneficial species, thereby leading to the incidence of systemic adverse effects. Specifically, the bacterial diversity of CRC patients following anticancer treatment seemed to be lower than that of nontreated patients, as indicated by the downregulation of Chao1 index, yet no statistical difference was detected [8,10]. However, in this study, the intestinal flora diversity, like Chao1 index, was obviously reduced in the fecal samples of anticancer-treated CRC patients (Figure 2), indicating a serious loss of bacterial species in CRC patients after treatment. In addition, the relative abundance of Firmicutes and Fusobacteria decreased at the phylum level, while the abundance of Bacteroidetes and proteobacteria increased in the fecal samples of CRC patients receiving radical surgery combined with adjuvant chemotherapy, when compared with that of preoperative samples [9]; this was consistent with our findings, as shown in Figure 3. Meanwhile, at the genus level, the relative levels of *Akkermansia*, *Faecalibacterium*, and *Sutterella* were found to be enlarged, as well as *Prevotella* and *Roseburia* were found to be reduced in chemotherapy-treated CRC patients, both in our study (Figure 3) and other studies [8,9,10]. In addition, there were some inconsistent observations on other bacterial genera such as *Bacteroides*, *Bifidobacterium*, *Lactobacillus*, and *Blautia* among these studies, which might be explained by the usage of different chemotherapy regimens, and whether or not surgery was performed. Altogether, these results indicate an alteration in the gut microbiota composition accompanied by surgery and chemotherapy for CRC patients.

Interestingly, applying probiotics to intervene in anticancer therapy-induced gut dysbiosis improves gastrointestinal adverse effects and has achieved significant effects [25,28]. Currently, *Lactobacillus* and *Bifidobacterium* are the most studied probiotics for preventing and controlling CRC in clinical and preclinical publications. In CRC patients, Minoru Mizuta and colleagues found that perioperative oral administration of *Bifidobacterium* may attenuate postoperative inflammation, and contribute to a balanced intestinal microbiota by increasing Actinobacteria and decreasing Firmicutes after colorectal resection [29]. Zhiguang Gao et al. demonstrated that surgical patients treated with a probiotic preparation containing live *B. longum*, *L. acidophilus* and *E. faecalis* species exhibited higher bacterial diversity and density compared to that of nontreated patients in the mucosa-adherent microbiota, as well as a significant reduction in *Fusobacterium* and expansion of *Enterococcus* and *Proteobacteria* [30]. In the animal CRC model, Lu Yuan et al. suggested that the administration of a probiotic combination containing *Lactobacillus* and *Bifidobacterium* could reduce the proportion of some opportunistic pathogens such as *Desulfovibrio* and *Escherichia-Shigella*, while enriching *Reseburia* and *Prevotella* genera in a CRC mice model following 5Fu-based chemotherapy [31]. A study performed by Ching-Wei Chang et al. also demonstrated that the oral probiotic *Lactobacillus casei* variety *rhamnosus* could prevent chemotherapy-induced intestinal mucositis and restore the disturbed gut microbiota composition, as indicated by increased Bacteroidetes and decreased Firmicutes at the phylum level in a CRC mice model [32]. However, upon chemotherapy treatment, these probiotics could not alter the antitumor efficacy of drugs. In addition, a few clinical studies focused on the potential effects of probiotics on chemotherapy-induced gut microbiota dysbiosis. In this study, we demonstrated that XELOX regimen-based chemotherapy significantly altered the gut microbiota composition in CRC patients, while probiotic administration could restore the disturbed gut microbiota without affecting the anticancer efficacy of chemotherapy, as shown by the results of blood routine analysis (Table S1).

Furthermore, specific changes in the gut metabolome have been presented in CRC subjects who underwent antitumor treatments, particularly in SCFAs [33,34]. Outside of regulating energy metabolism, SCFAs can also modulate the systemic immune response, inhibit the intestinal inflammatory reaction, and recover gut homeostasis for patients [13]. Here, we analyzed the concentration of SCFAs in the fecal samples among CRC patients receiving chemotherapy and a subsequent probiotic interventional treatment. Our results indicated that the contents of the main SCFAs such as acetic, propionic, and butyric acids were dramatically reduced with the course of chemotherapy, whereas taking probiotics was valid against these changing trends of SCFAs (Figure 5). The possible explanation is that the disturbed gut microbiota and intestinal mucositis by chemotherapy reduced the production of SCFAs, while probiotics and their recovered bacterial populations reversely promoted the yield of those SCFAs.

There are some limitations to this study. The study design and data analysis did not take into account the influence of gut microbiome heterogeneity caused by individual differences on the experimental results. For instance, it was unclear whether there was a difference in the gut microbiota between these two groups after randomization. Although it has been demonstrated that probiotic intervention perioperatively or postoperatively could restore surgery-induced gut dysbiosis in CRC patients [24,29,30], this study still needed to consider whether the difference in the fecal microbiota and the generated SCFAs of these two groups after chemotherapy was caused by probiotics, or by individual differences among patients. Therefore, it would be better to take the fecal samples collected from both groups before chemotherapy and use them as a control group for further analysis. Additionally, multi-omics (e.g., metagenomics and metabolomics) should be applied to deeply explore the underlying mechanisms of probiotics in translational medicine research.

## 5. Conclusions

**Figure 6:**
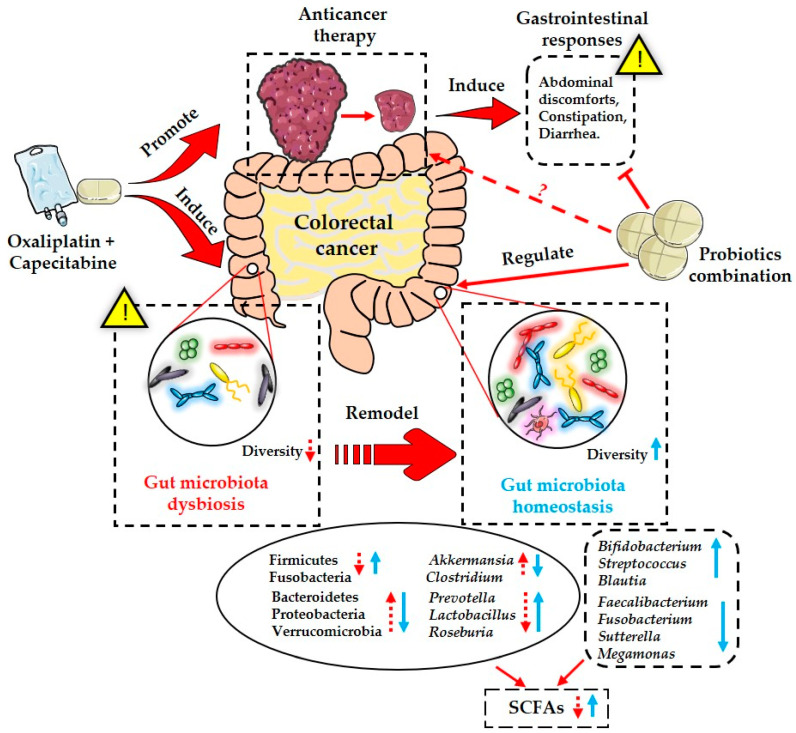
Schematic diagram of actions of probiotics combination in attenuating chemotherapy-induced gastrointestinal responses and gut microbiota dysbiosis.

In conclusion, the present study indicated that the administration of a probiotic combination containing four strains could significantly alleviate chemotherapy-induced gastrointestinal complications in CRC patients, including abdominal discomfort and diarrhea. This anticancer treatment, based on the XELOX regimen, also altered the structure and composition of the gut microbiota in CRC patients, as well the generation of fecal SCFAs (Figure 6). Specifically, probiotics protected against this chemotherapy-induced gut microbiota dysbiosis, and promoted the production of SCFAs (Figure 6). Although our finding added new evidence for the clinical application of probiotics to reduce the severity of gastrointestinal adverse effects caused by antitumor treatment, and to remodel the disturbed gut microbiota, more well-designed studies are urgent to further explore the underlying active mechanisms of probiotics. In addition, the standardized use of probiotics clinically, including the probiotic species, optimal dose, and duration of medication, should be more definitive in the future.

## Figures and Tables

**Table 1:** Participants’ characteristics.

Variables	Placebo Group (n = 50)	Probiotics Group (n = 50)	*p* Value
Gender (male/female)	29/21	24/26	0.316
Age (year)	62.1 ± 10.5	57.7 ± 11.9	0.053
Tumor category			0.326
T0	0	0	
T1	2	1	
T2	10	13	
T3	31	34	
T4	7	2	
Node category			0.614
N0	30	30	
N1	10	13	
N2	10	7	
Metastasis category			
M0	50	50	/
TNM category			0.891
I	8	6	
II	21	24	
III	21	20	
Location of tumor			0.603
Right colon	15	11	
Left colon	6	6	
Sigmoid colon	10	11	
Rectum	19	22	

**Table 2:** Comparison of incidence of gastrointestinal adverse reactions after chemotherapy.

Group	n	Nausea (%)	Acid Reflux (%)	Abdominal Pain (%)	Abdominal Distention (%)	Constipation (%)	Diarrhea (%)
Probio	50	11 (22.00)	2 (4.00)	3 (6.00)	5 (10.00)	4 (8.00)	8 (16.00)
Placebo	50	17 (34.00)	6 (12.00)	12 (24.00)	14 (28.00)	14 (28.00)	20 (40.00)
χ^2^		1.786	1.223	5.020	4.159	5.488	7.143
*p* value		0.181	0.169	0.025	0.041	0.019	0.008

## Data Availability

The data used to support the findings of this study are available from the corresponding author upon request.

## Supplementary Materials

The following supporting information can be downloaded at: https://www.mdpi.com/article/10.3390/nu15020356/s1, Figure S1: Comparison of the differences of α and β diversities among CRC patients; Figure S2: Spearman correlation analysis between SCFAs and the altered gut microbial genera; Table S1: Comparison of blood routine test between CRC patients in placebo and probiotics groups.
