# Supplementary material for: Postoperative Probiotics Administration Attenuates Gastrointestinal Complications and Gut Microbiota Dysbiosis Caused by Chemotherapy in Colorectal Cancer Patients
Source: Nutrients. 2023 Jan 11;15(2):356. doi: 10.3390/nu15020356 (PMC9861237; doi:10.3390/nu15020356)
Supplement: Supplementary file 1 [file nutrients-15-00356-s001.zip › Supplementary material-CRC-Li et al-202211.pdf]

Supplementary Figure S1

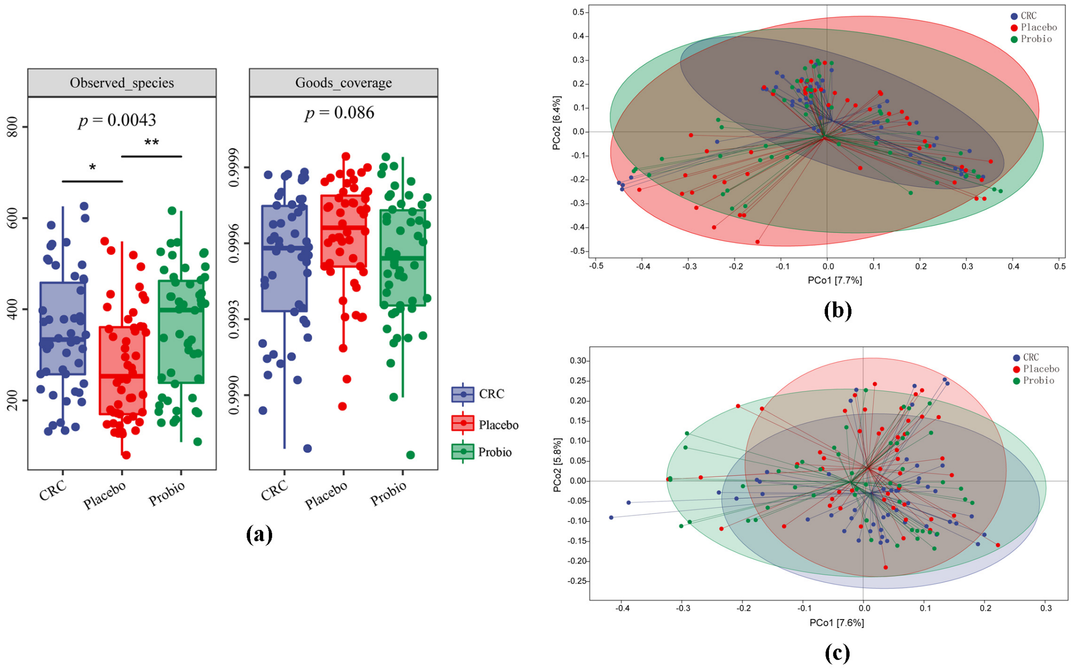

Figure S1. Comparison of the differences of α and β diversities among CRC 428 patients. (a) Observed species and Goods_coverage indexes of gut microbial α diversity in the fecal samples among the three groups. (b) and (c) The PCoA analysis of gut microbial β diversity based on Bray_curtis and Weighted_unifac distances, respectively. CRC, the standard reference group before anticancer treatment. Placebo, the control group taking placebo. Probio, the treatment group taking probiotics combination. Multiple comparison analysis based on Kruskal-Wallis test following dunn's test, * $p < 0.05$, ** $p < 0.01$.

Supplementary Figure S2

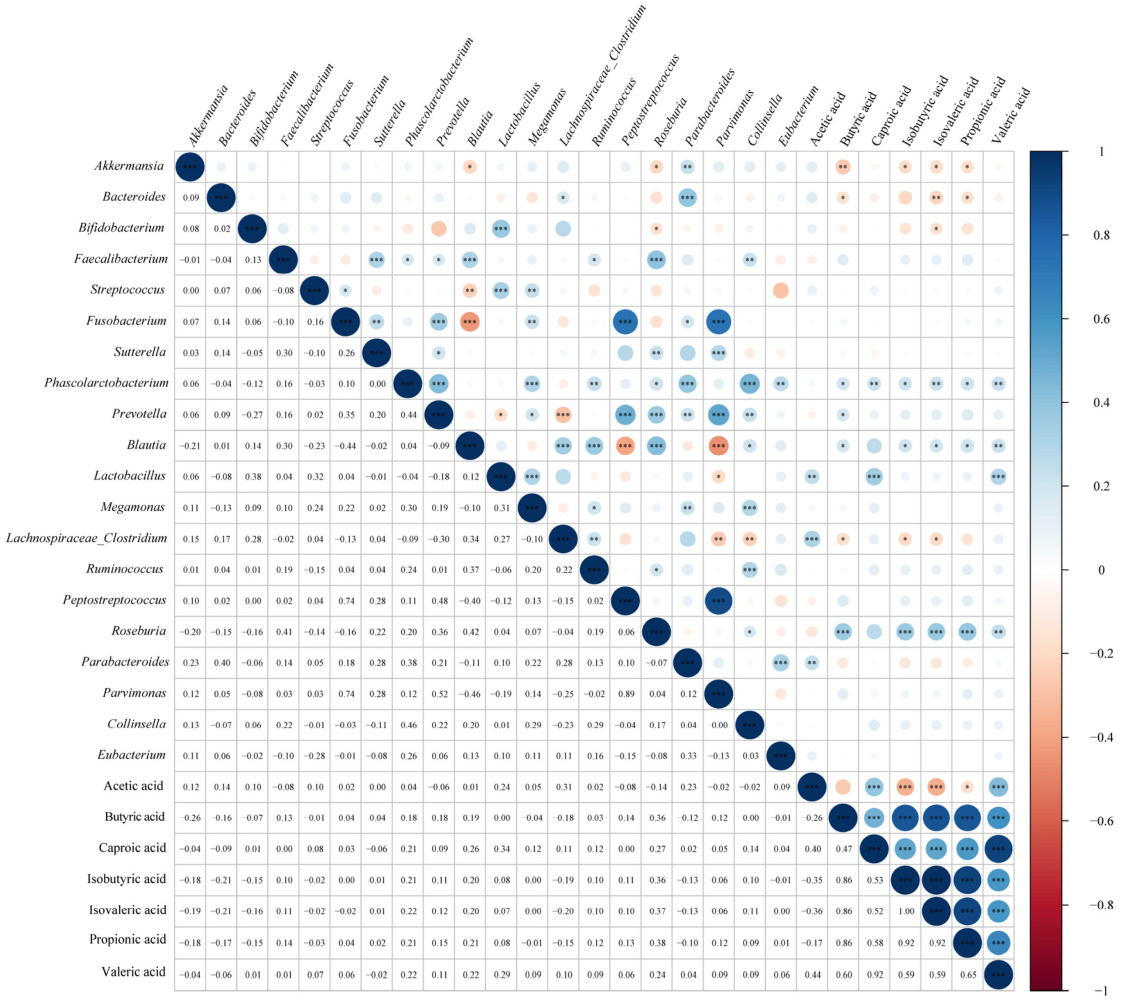

Figure S2. Spearman correlation analysis between altered gut microbes at the genus level and SCFAs. P values are depicted in red and blue, where red refers to a negative correlation and blue refers to a positive correlation. * $p < 0.05$; ** $p < 0.01$; *** $p < 0.0001$.
